# Retroperitoneal undifferentiated pleomorphic sarcoma with total nephrectomy: a case report and literature review

**DOI:** 10.3389/fsurg.2023.1166764

**Published:** 2023-06-15

**Authors:** Kai Yu, Lan Wang, Fan Bu, Jingxuan Zhang, Yubin Hai, Rui Hu, Ji Lu, Xiaoju Shi

**Affiliations:** ^1^ Department of Urology, The First Hospital of Jilin University, Changchun, China; ^2^ Department of Hepatobiliary and Pancreatic Surgery, The First Hospital of Jilin University, Changchun, China; ^3^ Department of Plastic and Aesthetic Surgery, The First Hospital of Jilin University, Changchun, China

**Keywords:** treatment, case report, pleomorphic undifferentiated sarcoma, diagnosis, retroperitoneum

## Abstract

**Background:**

Undifferentiated pleomorphic sarcoma (UPS) is a highly malignant soft tissue sarcoma with a poor prognosis and no clear effective clinical means for treatment, and there has been no significant progress in research within this field in recent years. This study aimed to investigate the epidemiology, etiology, clinical features, diagnostic modalities, various treatment modalities, and prognosis of retroperitoneal undifferentiated pleomorphic sarcoma and to contribute to the clinical management of this type of disease. In this study, we report a case of undifferentiated pleomorphic sarcoma with a primary origin in the retroperitoneum. Undifferentiated pleomorphic sarcoma occurring in the retroperitoneum is rarely reported.

**Case description:**

A 59-year-old man with abdominal distension and pain for 4 months presented to our hospital after the failure of conservative treatment. A 9.6 cm by 7.4 cm mass in the left retroperitoneum was found on a CT scan of the whole abdomen with three degrees of enhancement. After surgical treatment, the tumor and the left kidney were completely removed, and pathological examination and genetic sequencing showed an apparent undifferentiated pleomorphic sarcoma. The patient subsequently declined follow-up treatment and is currently alive and well.

**Conclusions:**

At the current level of clinical technology, the treatment of undifferentiated pleomorphic sarcoma is still in the exploratory stage, and the scarcity of clinical cases of this disease may have hindered the acquisition of clinical trials and research data for this disease. At present, the first choice of treatment for undifferentiated pleomorphic sarcoma is still radical resection. In the existing clinical studies, there are no strong data to support the effect of preoperative neoadjuvant chemoradiotherapy and adjuvant chemoradiotherapy in clinical practice. Similar to other diseases, the use of radiotherapy and chemotherapy before and after surgery may be a potential treatment for this disease in the future. Targeted therapy for this disease still needs further exploration, and we need more reports on related diseases to promote future treatment and research on this disease.

## Introduction

Undifferentiated pleomorphic sarcoma (UPS), a malignant soft tissue tumor of mesenchymal origin, occurs in middle-aged and elderly people, more often in men than in women. The disease occurs mainly in the extremities and only rarely in the retroperitoneum (1, 2), and its pathogenesis is unclear. The disease was first reported in 1964 as malignant fibrous histiocytoma (3), which has since been renamed undifferentiated pleomorphic sarcoma (4). The occurrence of this tumor in the retroperitoneum is less reported, and only eight cases have been described, of which five tumors originated in the kidney (5–9), two were documented in a clinical study by A. Pirayesh (10), and one was a paraspinal primary (11). We report a case of undifferentiated pleomorphic sarcoma arising in the retroperitoneum in a 59-year-old man and review the available medical literature on undifferentiated pleomorphic sarcoma to summarize the epidemiology, etiology, clinical presentation, radiologic features, diagnosis, and treatment options, including radiotherapy, chemotherapy, and targeted therapy for this rare tumor.

## Case report

### History and examination

The patient, 59 years old, had pain in the left upper abdomen after eating before April and lost 15 kg in the past 2 months, with no relief after taking oral gastric medication. In order to seek further treatment, he consulted our outpatient clinic.

### Imaging findings

**Figure 1:**
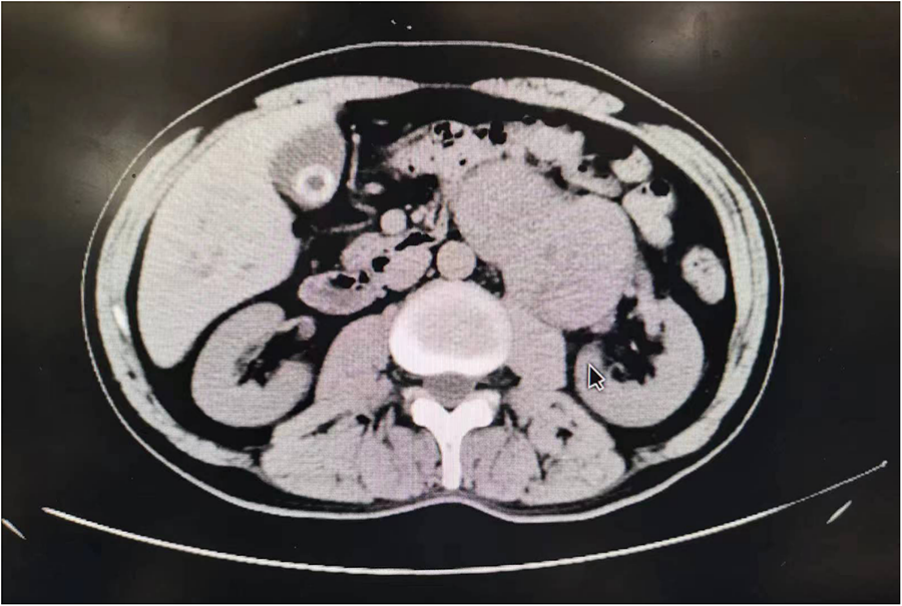
The patient's retroperitoneal undifferentiated pleomorphic sarcoma.

A mass-like, dense soft tissue shadow was seen in the left upper abdomen, measuring approximately 9.6 cm by 7.4 cm, with a CT value of about 41 HU. On enhancement scan, the mass was inconsistent enhancement and poorly demarcated from the tail of the pancreas, the left adrenal gland, and the left kidney, and the pancreatic duct was not dilated. Intraoperatively, the patient's mass was found to be located on the dorsal side of the pancreas, adjacent to the abdominal aorta and to the right of the splenic hilum, with unclear demarcation from the left kidney, as shown in Figure 1.

### Surgery

**Figure 2:**
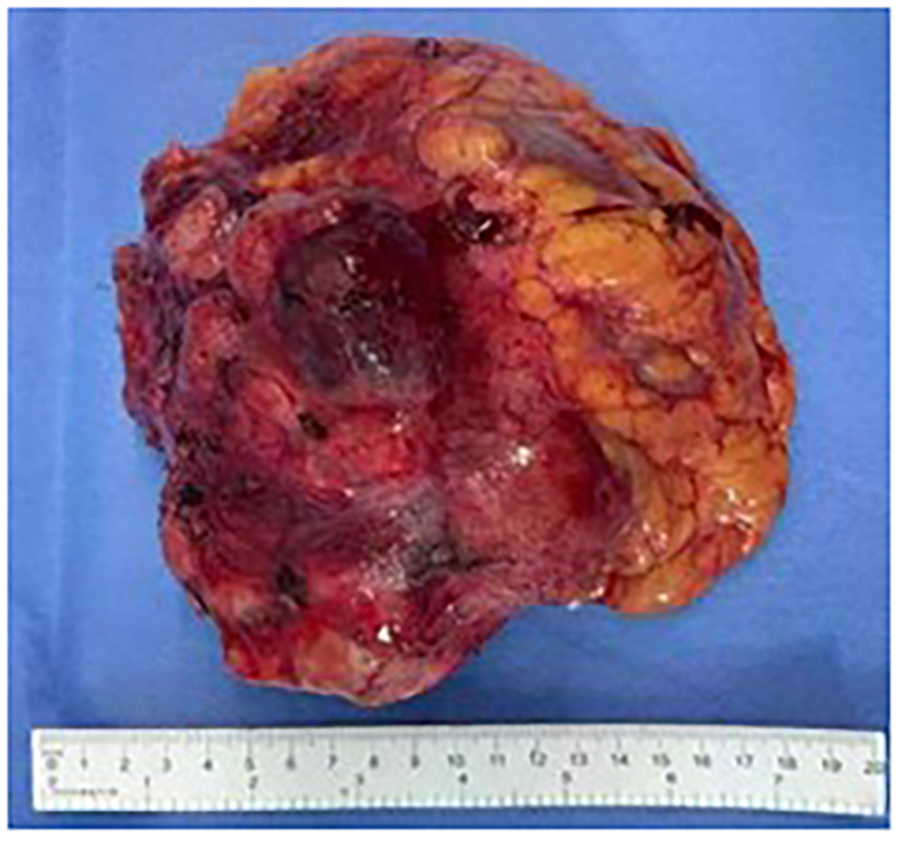
Gross observation of undifferentiated pleomorphic sarcoma.

The patient's preoperative imaging tended to indicate malignancy, and the tumor had certain boundaries with the kidney, but the anatomical location of the tumor was complicated, and the physician suggested a puncture biopsy. However, the family refused, considering factors such as possible kidney injury and bleeding, so this preoperative procedure was not performed. The general appearance of the tumor was observed intraoperatively, and it was found to be soft and irregular in shape, adhering more closely to the renal vessels, which made separation difficult. To achieve a complete resection of the tumor and to reduce the possibility of tumor recurrence after surgery, it was therefore decided to remove the ipsilateral kidney intraoperatively. The patient was preoperatively diagnosed with a retroperitoneal mass and underwent a combined left nephrectomy, left adrenalectomy, and mass removal. The tumor was solid, bloody yellowish, and the visible perirenal fat capsule was largely visible as shown in Figure 2.

### Histopathologic findings

**Figure 3:**
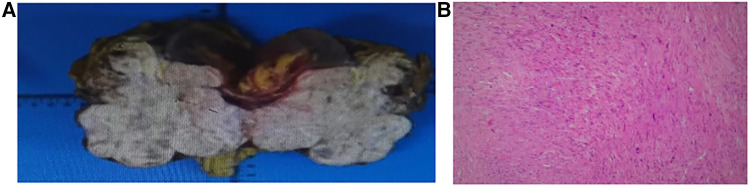
(**A**) Macroscopic tumor. (**B**) HE staining of tumor.

The pathological findings were undifferentiated pleomorphic sarcoma, not excluding dedifferentiated liposarcoma, as jointly diagnosed by two senior pathologists at our hospital. The left kidney and retroperitoneal mass had a total weight of 816.5 g. The volume of the mass was 12.0 cm by 9.5 cm by 6.5 cm, the surface was slightly stringy, the cut surface was grayish white, firm, and hard, and two tough nodules, both 0.3 cm in diameter, were palpated in the adipose tissue around the mass. The renal pelvis and ureteral mucosa were not abnormal. No lymph nodes were seen in the renal hilum. The adrenal gland was 4.5 cm by 1.0 cm by 0.8 cm in size, golden yellow in color, firm, and tough in texture. The tumor volume was 12 cm by 9.5 cm by 6.5 cm, and the nuclear schizophrenia was about 10 nuclei/10 HPF. Local tumors necrosis, local invasion of the adrenal parenchyma, and renal peritoneal adhesions did not invade the renal parenchymal vasculature and nerves, tumor infiltration of the ureter and blood vessels were visible, and tumor infiltration was visible at the end of the cut edge. A metastasis was visible in the lymph nodes around the tumor (3/3), as shown in Figure 3A.

Immunohistochemistry: CD34(−), Desmin(−), Ki-67(+40%), SMA(partial +), S-100(scattered +), CD117(−), Dog-1(−), HMB45(−). TFE3(+), CK-pan(−), H-caldesmon(−), β-catenin(membrane +). STAT6(−), CK-pan(−), Vimentin(+), CK5/6(−), WT-1(weak+), Calretinin(−), D2-40(−).

### Examination findings

Diagnosis: retroperitoneal (including left kidney) malignant tumor of mesenchymal origin consistent with undifferentiated pleomorphic sarcoma; it was recommended that genetic testing was attempted to further exclude dedifferentiated liposarcoma, as shown in Figure 3B.

### Gene sequencing

The tumor mutational burden (TMB) was 2.922.92 Muts/Mb (ModerateModerate), microsatellite instability (MSI) was detected as microsatellite stable (MSS), positive gene (1): TP53 p.I332M; negative gene (1): PTEN copy number was decreased in tumor tissues. The HLA-I-like molecular genotype was detected as HLA-I (A, B, C); heterozygous secondary variants were detected as PTEN copy number reduction only; the evidence level was C, which may be sensitive to platinum-based chemotherapy modalities; and no hereditary tumor-related genetic variants were detected. The genetic diagnosis also excluded the possibility of dedifferentiated liposarcoma.

### Postoperative course

The patient recovered well after surgery with only transient renal insufficiency. After consultation with the Department of Nephrology and the Department of Urology I, relevant symptomatic treatment was performed, and the patient was discharged 7 days after the surgical intervention, with the renal function returning to normal at the time of discharge. After the postoperative joint consultation with several specialists, the combination of the targeted therapy drug anlotinib and the immunotherapy drug pablizumab was recommended. The patient was informed about his condition but did not indicate his attitude toward the next treatment. However, according to the current follow-up results, the patient is currently in good health with no significant abnormalities, and we are continuing to follow up with him.

## Discussion

### Epidemiology and etiology

Soft tissue sarcomas (STSs) are rare malignant tumors of mesenchymal origin that account for approximately 1% of adult malignancies (12), with a large number of approximately 50 subtypes, of which the less differentiated ones that can exhibit multiple cellular forms are called undifferentiated pleomorphic sarcomas. Undifferentiated pleomorphic sarcomas can occur in any part of the body, with the extremities being the most common site (50% in the lower extremities and 20% in the upper extremities) (13), with only a few occurring in the retroperitoneum. This case is that of a 59-year-old man with undifferentiated pleomorphic sarcoma occurring in the retroperitoneum, which is consistent with the age (around 60 years) and gender (male) of onset reported in the literature (13). The incidence of the disease is extremely low, but there is an increasing trend year by year, with only three cases per 100,000 in 2013 (14) and three cases per 45,000 to date (15). Metastatic foci of the disease are mostly found in the lungs and, to a lesser extent, in the liver (16). The odds ratio (OR) of smoking for UPS is 2.05 (95% confidence interval, 1.78–2.37; *p* < 0.01) (17), and more than 30% of patients with undifferentiated pleomorphic sarcoma were found to have a family history in a previous study (18, 19). Approximately 3%–5% of patients may develop locally more harmful RA-UPS as a result of radiation therapy, which is commonly referred to as radiation-associated undifferentiated pleomorphic sarcoma (RA-UPS) (20, 21).

### Clinical presentation

Patients with undifferentiated pleomorphic sarcoma usually have no obvious specific symptoms, and clinical symptoms are usually related to the location and size of the tumor.

Patients typically present with clinical indications that are signs of tumor compression. In this case, the patient presented with gastritis because the mass was located in the posterior peritoneum and pressed forward on the stomach, and some patients may report to the clinic with respiratory symptoms due to tumor metastasis in the lungs. In similar cases and this case report, common symptoms included abdominal pain (lumbago) in five cases (83.3%) (5, 6, 8, 9), weight loss in four cases (66.7%) (5, 6, 8), anemia in three cases (50%) (6, 8, 9), fever in two cases (33.3%) (8, 9), and difficulty urinating in one case (16.7%) (8).

### Diagnosis and radiological characteristics

Radiological details about UPS are scarce in the literature because of how uncommon it is. Imaging of this disease is usually non-specific, and exclusionary diagnostic methods are mostly used (22). The majority of well-differentiated liposarcomas contain adipose tissue within the tumor, and some poorly differentiated liposarcomas have calcifications (23); smooth muscle sarcomas have vascular invasion and hemorrhagic necrosis as the main imaging features (24); and undifferentiated pleomorphic sarcomas have calcifications that typically appear at the edge of the lesion (23). Imaging is of great importance for surgical treatment, and normally the surgical margins are selected 2–3 cm outside the tumor infiltration shown on imaging; with the development of imaging techniques, the preoperative diagnosis and postoperative pathologic diagnosis of the extent of infiltration have become more consistent. A “tail sign”, a frequent curvilinear signal extension of the mass, can be found in some MRIs of infiltrative UPS and can be used as a potential diagnostic basis for the disease (25, 26). However, generally speaking, the diagnostic imaging modality is more limited in its ability to identify the disease. The main gold standard for the diagnosis of this disease is still pathology, and imaging is typically only used as a reference and for auxiliary evaluation. In pathological examination, care should be taken to protect the specimen and to avoid cross-sectioning, which may affect the assessment of tumor depth and infiltration. There are no specific histochemical markers for undifferentiated pleomorphic sarcoma in clinical practice, and positive or negative endosialin (27) is commonly used as a basis for diagnosis.

### Treatment

Due to the rarity of UPS, standard management guidelines have not yet been established. Despite a multimodal approach including surgery, radiotherapy, and chemotherapy, targeted therapy is the predominant therapy. In addition, surgical resection is the leading treatment for performing gross tumor resection (GTR), and the achievement of negative surgical margins due to the extension of the resection is one of the most frequently reported predictors of recurrence and survival. Surgical treatment has removed the gross tumor cells, but potentially smaller lesions or tumor cells in circulating cells are still alive, and a combination of other treatments is needed to effectively control tumor recurrence.

### Radiotherapy

Regarding the effect of radiation therapy, most researchers believe that local radiotherapy is more effective in tumors that have not infiltrated. Especially in undifferentiated pleomorphic sarcomas located in the extremities and superficial occurrences (28), radiotherapy can induce increased antigen expression at the tumor site, promote immune cell infiltration and antigen cross-presentation, and, to some extent, alter the tumor microenvironment, thereby affecting tumor cell proliferation (29, 30). However, no significant effect has been seen in tumors with infiltrative metastases (31, 32). In our patient, radiotherapy was often ineffective because the patient's tumor was located in the retroperitoneum, as per the observations in previous studies (33).

As for the timing of radiation therapy application, some studies have shown that radiation therapy in the perioperative period, preoperatively, can prolong the patient's survival time (34). With respect to the type of radiation therapy used, heavy ion radiation therapy, which can cause irreparable DNA cluster damage in tumor cells, has gradually become a key technology in tumor radiation (35), which was better validated in Zaixing Wang's study. At the same time, however, we need more randomized controlled trials for in-depth studies on the development of technology in this field (36). However, radiation therapy also has certain side effects, and some patients have developed RA-UPS after receiving radiotherapy, which has an incidence of about 0.16% (37) and a poor prognosis. Patients should receive radiation therapy with controlled radiation doses to avoid the occurrence of RA-UPS as much as possible.

### Chemotherapy

For the treatment of deep tumors in several parenchymal organs and RA-UPS, chemotherapy performs better compared to radiation therapy (38). The effectiveness of chemotherapy in this disease is controversial, and there are no clear clinical guidelines for its description. Different case reports and studies have shown that chemotherapy may extend the survival time of patients to some extent (39, 40). In the application of chemotherapeutic agents, the effectiveness of adriamycin alone is still being investigated (41), and the combination of adriamycin with cyclophosphamide is still the drug of choice for chemotherapy of this disease in clinical practice (42). In a study by Paul Lorigan, the combination of doxorubicin and cyclophosphamide was also employed, with comparatively good results (43). Due to the large size of the tumor and the structural similarity of the core to the central region of the parenchymal organ, chemotherapy is typically more effective in larger tumors (>8 cm) (44), and secondary tumor side effects are less severe than with radiation (45). In studies on the timing of drug application, neoadjuvant chemotherapy before surgery can also be used with good results (46).

### Targeted therapy

Undifferentiated pleomorphic sarcoma (UPS) is an aggressive adult soft tissue sarcoma characterized by low tumor mutational burden (TMB) and high copy number alterations (47). In recent years, PD-1 and PD-L1 have been identified as novel antitumoral targets. PD-1/PD-L1 interaction is the main pathway of immune control of tumor suppression, and PD-1 has gradually become a hot topic for research (48). PD-1/PD-L1-related immune responses are more common in UPS (49, 50), but more as a differential diagnosis, one of the methods that has limitations for the prognosis prediction of the disease (51). In a study by YangYou et al., anti-angiogenesis inhibitors combined with PD-1 inhibitors had a good effect on UPS (52), and in a study by Zhichao Tian et al., paclitaxel combined with PD-1 inhibitors also had a significant effect on UPS (53), but some patients had poor results with PD-1 inhibitors (54). PD-1 may be a potential therapeutic target for UPS in the future, and its expression is important in influencing CD8+ T lymphocyte infiltration and patient prognosis (55). Using a comparative oncology approach, researcher Ashley M. Fuller identified DNMT3B, which leads to DNA methylation patterns in human undifferentiated pleomorphic sarcoma, as a potential therapeutic target (56). However, anti-methylation drugs currently in clinical use have not yet been able to provide effective treatment for this disease due to poor drug uptake or systemic toxicities, and future studies on hENT1 (SLC29A1) may enhance drug uptake to treat the disease (57). Christina L. Roland found that cyclin D1, pEGFR, pIGF-1R, and PTEN deletion (*p* < 0.001) and AXL overexpression (*p* = 0.015) were associated with reduced disease-specific survival (DSS) (58). In addition, neurotensin receptor 1 (NTSR1) (59), anti-human tumor endothelin 1 (TEM-1) (60), and various other targeted therapeutic targets are under progressive research by related scholars and may become effective for the treatment of this disease in the future.

### Prognosis

The survival rate of this condition is related to the site of disease onset, with a 5-year survival rate of more than 70% for tumors in the trunk and extremities and less than 50% for tumors in the head and neck (61), which may be related to the richer blood supply and more important anatomical sites in the head and neck. Currently, the American Joint Committee on Cancer (AJCC) staging system (62) is used to evaluate the main clinical prognosis of UPS, and patient prognosis is mainly related to recurrence and distant metastases. Among them, tumor recurrence is mainly related to size (>5 cm), tissue infiltration (>5.5 mm), and whether the margins are positive, with more than 30% of patients likely to experience recurrence (63, 64). The presence of a “tail sign” in preoperative imaging may also represent a high likelihood of recurrence (64). Risk factors for distant metastases are related to the tumor site (trunk and extremities), tumor size (>2 cm), and infiltration (invasion of subcutaneous fat and lymphatic vessels). Risk factors for all-cause mortality were gender (male), ethnicity (white), age (>55 years), immunosuppression, tumor size >2 cm, and lymphovascular invasion (65–67). However, the prognosis of patients with this disease is usually highly variable, and a prognostic evaluation criterion called the nomogram is emerging as a novel method for the evaluation of this disease (13, 68). Radiation-associated undifferentiated pleomorphic sarcoma patients usually have a worse prognosis (69), and the disease's strong PD-1 expression in immunohistochemistry represents a poor prognosis and IDO-1 expression a better prognosis (70).

## Conclusion

In the patient described here, the tumor adhered to the renal hilum and adhered more tightly to the renal vessels, making separation more difficult and showing obvious infiltration. There were some surrounding lymph node metastases, but no distant metastases for the time being. The tumor, left kidney, and left adrenal gland were completely removed during surgery. After follow-up, the patient is in good mental and physical condition but did not express a clear opinion on the future treatment.

In conclusion, retroperitoneal undifferentiated pleomorphic sarcoma is rare and difficult to diagnose. There are no obvious specific signs in the early clinical stage, and most patients come to the hospital with non-specific symptoms such as tumor compression. For this disease, we should classify and summarize the imaging manifestations and actively perform tumor puncture biopsy if the benign and malignant tumors cannot be clearly determined. In accordance with the pathological results of tumor puncture, relevant radiotherapy and chemotherapy should be performed during the perioperative period to reduce the risk of surgery and postoperative recurrence. Currently, there are no clear guidelines for the treatment of this disease, and more in-depth research is needed regarding chemotherapy and comprehensive treatment.

## Data Availability

The raw data supporting the conclusions of this article will be made available by the authors, without undue reservation.
